# An MHC-I Cytoplasmic Domain/HIV-1 Nef Fusion Protein Binds Directly to the μ Subunit of the AP-1 Endosomal Coat Complex

**DOI:** 10.1371/journal.pone.0008364

**Published:** 2009-12-18

**Authors:** Rajendra Kumar Singh, David Lau, Colleen M. Noviello, Partho Ghosh, John C. Guatelli

**Affiliations:** 1 Department of Medicine, University of California San Diego, La Jolla, California, United States of America; 2 Department of Chemistry and Biochemistry, University of California San Diego, La Jolla, California, United States of America; 3 The San Diego Veterans Affair Healthcare System, La Jolla, California, United States of America; Institut Pasteur Korea, Republic of Korea

## Abstract

**Background:**

The down-regulation of the major histocompatibility complex class I (MHC-I) from the surface of infected cells by the Nef proteins of primate immunodeficiency viruses likely contributes to pathogenesis by providing evasion of cell-mediated immunity. HIV-1 Nef-induced down-regulation involves endosomal trafficking and a cooperative interaction between the cytoplasmic domain (CD) of MHC-I, Nef, and the clathrin adaptor protein complex-1 (AP-1). The CD of MHC-I contains a key tyrosine within the sequence YSQA that is required for down-regulation by Nef, but this sequence does not conform to the canonical AP-binding tyrosine-based motif Yxxφ, which mediates binding to the medium (μ) subunits of AP complexes. We previously proposed that Nef allows the MHC-I CD to bind the μ subunit of AP-1 (μ1) as if it contained a Yxxφmotif.

**Methods and Findings:**

Here, we show that a direct interaction between the MHC-I CD/Nef and μ1 plays a primary role in the down-regulation of MHC-I: GST pulldown assays using recombinant proteins indicated that most of the MHC-I CD and Nef residues that are required for the down-regulation in human cells contribute to direct interactions with a truncated version of μ1. Specifically, the tyrosine residue of the YSQA sequence in the MHC-I CD as well as Nef residues E62-65 and P78 each contributed to the interaction between MHC-I CD/Nef and μ1 *in vitro*, whereas Nef M20 had little to no role. Conversely, residues F172/D174 and V392/L395 of the binding pocket on μ1 for Yxxφ motifs were required for a robust interaction.

**Conclusions:**

These data indicate that the MHC-I cytoplasmic domain, Nef, and the C-terminal two thirds of the μ subunit of AP-1 are sufficient to constitute a biologically relevant interaction. The data also reveal an unexpected role for a hydrophobic pocket in μ1 for interaction with MHC-I CD/Nef.

## Introduction

Human immunodeficiency virus type 1 (HIV-1) Nef is a 27 kDa protein with no known enzymatic activity that appears to function by mediating protein interactions. Nef contributes to high levels of replication and the pathogenesis of primate lentiviruses such as HIV-1 and Simian Immunodeficiency Virus. Nef is expressed in abundance early during the viral replication cycle, and it down-regulates major histocompatibility complex class I (MHC-I) glycoproteins from the surface of infected cells. Nef has several functions in addition to the down-regulation of MHC-I molecules [1], such as the removal of CD4 from the surface of infected cells [2], [3]. The Nef-mediated down-regulation of MHC-I and CD4 is dependent on specific sequences in the cytoplasmic domains of these target proteins [4], [5]. In the case of MHC-I, Nef induces not only internalization from the plasma membrane but also the retention and degradation of MHC-I within the endo-lysosomal system [6]. Nef also affects signaling through the T-cell receptor CD3 in T-lymphocytes [7]. To perform these functions, Nef associates with the plasma membrane and various endosomal membranes via N-terminal myristoylation [8].

Membrane trafficking within the endosomal system is mediated in part by vesicles coated with adaptor protein (AP) complexes. The AP family consists of four members: adaptor protein complex-1 (AP-1), adaptor protein complex-2 (AP-2), adaptor protein complex-3 (AP-3), and adaptor protein complex-4 (AP-4). AP-1 and AP-2 (as well as AP-3 and AP-4) are heterotetramers. AP-1 contains the four subunits γ, β1, μ1, σ1, whereas AP-2 contains α, β2, μ2, σ2. These complexes localize to different membranes: AP-1 is found on the membranes of the *trans*-Golgi network (TGN) and endosomes, whereas AP-2 is found on the plasma membrane [9]. The AP complexes recruit scaffolding proteins, such as clathrin, as well as cargo proteins, including transmembrane receptors and proteins that are resident throughout the endosomal system.

AP-1 is a key cellular protein complex through which Nef mediates the down-regulation of MHC-I [10]. Two distinct binding sites are present on the AP-1 complex for target proteins: a site on the “hemi-complex” formed by the γ and σ subunits that binds to dileucine-based signals which conform to the sequence E/DxxxLφ, and a site on the μ1 subunit that binds to tyrosine-based signals which conform to the sequence Yxxφ, where φ represents a bulky hydrophobic residue [11], [12]. The molecular basis for the binding of tyrosine-based and dileucine-based motifs to AP complexes has been explained by X-ray crystal structures [11], [12]. The crystal structure of the AP-2 μ subunit in complex with the peptide FYRALM reveals a “two prong in socket” mechanism, in which one prong is the tyrosine residue of the Yxxφ sequence and the other prong is the hydrophobic residue at the Y+3 position [11]. Recently, the crystal structure of the AP-2 complex with a peptide from CD4 containing a dileucine-based motif revealed the basis for this interaction [12]. Biochemical analyses further revealed a cooperative interaction between the cytoplasmic domain of CD4, Nef, and the σ2/α hemicomplex [13].

MHC-I, Nef and AP-1 appear to participate in a cooperative interaction in which the MHC-I molecule and Nef together form a novel ligand for AP-1 [14]. The molecular basis of this interaction is poorly understood: the minimal components are not defined, and its structural basis is unknown. Notably, the cytoplasmic domain (CD) of MHC-I contains a key tyrosine within the sequence YSQA that is required for down-regulation by Nef. However, this sequence does not conform to the canonical AP-binding tyrosine-based motif Yxxφ.

To identify the minimal components sufficient for the interaction between the MHC-I CD, Nef, and AP-1, the relevant proteins were expressed recombinantly in *E. coli*, purified, and used for GST pull down assays. In our experimental design, we used a chimeric protein in which full-length Nef was fused to the C-terminus of the cytoplasmic domain of MHC-I (MHC-I CD-Nef). We also used two N-terminal truncation proteins of μ1: μ121 (residues 121–423) and μ158 (residues 158–423). We used truncated forms of μ1 in our experiments, because of their better expression and solubility than the full length μ1 (residues 1–423)**.** GST pull down assays demonstrated the formation of a complex between MHC-I CD-Nef and μ121 or μ158. Furthermore, the tyrosine within the MHC-I cytoplasmic sequence YSQA and key residues within Nef that are important for the down-regulation of MHC-I were required for this interaction. Finally, residues F172, D174, V392 and L395, which line the tyrosine motif-binding pocket in μ1, were also required. These data indicate that a direct interaction with μ1 explains most of the previously defined genetic determinants for the down-regulation of MHC-I by Nef. The data also suggest that a currently unidentified residue within either Nef or the MHC-I CD binds a hydrophobic pocket on μ1.

## Materials and Methods

### Antibodies and Reagents

Ni-resin was purchased from Sigma (St. Louis, MO) and GSTrap columns were purchased from GE Healthcare (Piscataway, NJ). Anti-His6-peroxidase mouse monoclonal antibody (Clone His-2) for the detection of His6-tagged recombinant proteins was purchased from Roche (USA Cat. No.04905270001). Sheep anti-Nef antiserum was raised against recombinant protein made in *E.coli* and provided by Dr. Celsa Spina (UCSD). Mouse anti-β- actin antibody was purchased from Sigma (St. Louis, MO). TOPO-TA cloning and Quickchange kit for site directed mutagenesis were purchased from Invitrogen (San Diego, USA) and Stratagene (San Diego, USA), respectively.

### Flow Cytometry

CEM T cells (5×10^6^) were cotransfected according to the manufacturer's instructions, using an AMAXA Nucleofector (Lonza Cologne AG) system at a concentration of 5×10^6^ cells/ml and 11 µg of plasmid DNA. The plasmid DNA mix contained 1 µg of pCG-GFP (a transfection reporter gene) and 10 µg of one of the following pCIneo-based CD8- fusion constructs: CD8-Nef, CD8-Nef LL/AA, CD8-CD-Nef LL/AA, CD8-CD (Y320A)-Nef LL/AA and CD8-CD (in which “-CD” indicates the cytoplasmic domain of MHC-I A2) and “CD8” indicates the luminal and transmembrane domains of CD8. Cells were incubated at 37°C overnight, stained with mouse anti-human CD8 antibody conjugated with phycoerythrin (PE; BD Pharmigen), and subsequently analyzed by two-color flow cytometry; a PE conjugated isotype control antibody was used to set the gate for CD8-positive cells, and untransfected cells were used to set the gate for GFP-positive cells.

### Cloning, Expression and Purification of MHC-I CD and Nef and Their Mutants

The sequence encoding HIV-1 Nef was previously fused to the amino acid sequence of MHC-I cytoplasmic domain using PCR and then inserted into the pGEX-4T1 vector, adding a GST tag at the N-terminus end of the protein [14]. Similarly, an MHC-I CD-Nef LL/AA mutant was previously cloned into the pGEX-4T vector, and the mutations within this sequence are as described [14]. MHC-I CD-only and Nef only GST-fusion proteins were cloned similarly [14]. These vectors were transformed into *E. coli* strain BL21 (DE3) pLysS. The *E. coli* cells were grown overnight at 37°C in 10 ml of LB medium containing ampicillin (100 µg/ml). Cells were then inoculated into 500 ml of fresh LB medium containing ampicillin and agitated until the culture density reached an OD_600_ of 0.6. The protein was over-expressed at room temperature by the addition of isopropyl β-d-thiogalactopyranoside (IPTG) to a final concentration of 1 mM. After overnight induction, the bacterial cells were harvested by centrifugation at 5000 rpm for 10 minutes at 4°C. Wet cell pellets were resuspended in 20 mM Tris HCl, pH 7.5 containing 150 mM NaCl and lysed in buffer containing 50 mM Tris HCl, pH 8.0, 150 mM NaCl, 5 mM EDTA, 10 mM MgCl_2_, 1 mM dithiothreitol (DTT), protease inhibitor cocktail (Roche) and 1% (v/v) Triton X-100 for 3 hrs at 4°C. Crude cell lysates were centrifuged at 13,000 rpm for 1 hr at 4°C. The soluble fractions were loaded onto GSTrap 1 ml HP columns pre-equilibrated with 50 mM Tris HCl, pH 8.0 and 150 mM NaCl; the columns were extensively washed with same buffer and then eluted using 50 mM Tris pH, 8.0 containing 10 mM reduced glutathione. The purity of the protein samples was analyzed using 12% SDS-PAGE. Similarly, mutants encoding Y320A in the MHC-I CD, and M20A, E62-65A, and P78A in Nef were cloned previously [14], and expressed in *E. coli* strain BL 21 (DE3) pLysS cells. All mutant proteins were purified using GSTrap columns.

### Cloning, Expression and Purification of the AP-1 Medium Subunit (μ1) and Related Mutants

A plasmid encoding AP-1 medium subunit (μ1) was obtained from Dr. Juan Bonifacino (NIH). PCR primers with the restriction sites *Nde*I and *Bam*HI appended were used to clone three μ1 constructs: μ full-length (1–423), μ121 (121–423) and μ158 (158–423). These truncations were designed based on previous constructs expressing variants of the highly related μ2 subunit, which has been crystallized in complex with a peptide containing a canonical YxxL motif [11]. These μ1 sequences were ligated using the *Nde*I and *Bam*HI sites into the expression vector pET15b, which introduces a His6-tag at the N-terminus. Proteins were expressed in *E. coli* BL21 (DE3) pLysS cells as described above for the MHC-I CD-Nef GST-fusion constructs. Soluble protein was obtained in the case of the μ1-deletion constructs μ121 and μ158. These two μ1 proteins were then purified using Ni-column affinity chromatography: supernatants after the lysis were loaded onto a His trap Ni-column; the column was washed with a buffer containing 20 mM Tris HCl pH, 7.5, 150 mM NaCl and 10 mM imidazole to remove unbound proteins; and then bound proteins were eluted using 20 mM Tris HCl, pH 7.5, 150 mM NaCl and 500 mM imidazole. The purity of the μ1 containing fractions was checked using 12 % SDS-PAGE. Similarly, we cloned and expressed the μ1 mutant F172A, D174S, V392A, and L395A. All μ1 mutants were made using the QuickChange kit (Stratagene) and confirmed by DNA sequencing. The mutant proteins were expressed and purified as described above for the μ121 and μ158 proteins.

### Glutathione S-Transferase (GST) Pulldown Experiments

We performed binding studies in vitro using partially purified recombinant proteins expressed in *E. coli* as described above. These partially purified proteins were combined and incubated overnight at 4°C. The next day, GST pulldowns were done by loading the mixed proteins onto GSTrap 1 ml HP-columns. Unbound proteins were removed by extensive washing using 50 mM Tris pH, 7.5 and 150 mM NaCl buffer. The bound fractions were eluted using buffer containing 50 mM Tris pH, 8.0 and 10 mM reduced glutathione, then analyzed on SDS-PAGE through Coomassie blue staining and/or Western blot.

### Western Blot

Protein samples were suspended in gel loading buffer, boiled and run on 12% SDS-PAGE. The proteins were transferred to a polyvinylidene difluoride membrane (PVDF) membrane. Membranes were blocked with 4% milk (w/v) in PBS-T buffer (0.02% v/v Tween 20) for an hour. Anti-His6-peroxidase mouse monoclonal antibody (1∶4000) was added in 1% milk+PBS-T for 1.5 hr. In some experiments, anti-Nef antibody (1∶1000) and anti-βactin antibody (1∶1000) were used, followed by appropriate peroxidase conjugated secondary antibodies. After four washes with PBS-T, proteins were detected using ECL reagent (GE Healthcare). In some case the blots were subsequently stained with Ponceau red to directly visualize the proteins.

## Results and Discussion

### Minimal Constituents of a Complex between the MHC-I Cytoplasmic Domain, Nef, and μ1 *In Vitro*

**Figure 1:**
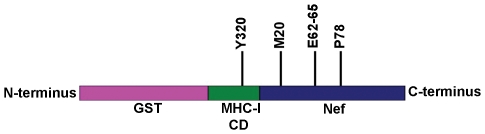
Schematic representation of the GST-MHC-I CD-Nef fusion protein. Key residues in MHC-I CD (Y320) and HIV1-Nef (M20, E62-65, P78) used in mutational and binding studies are shown.

**Figure 2:**
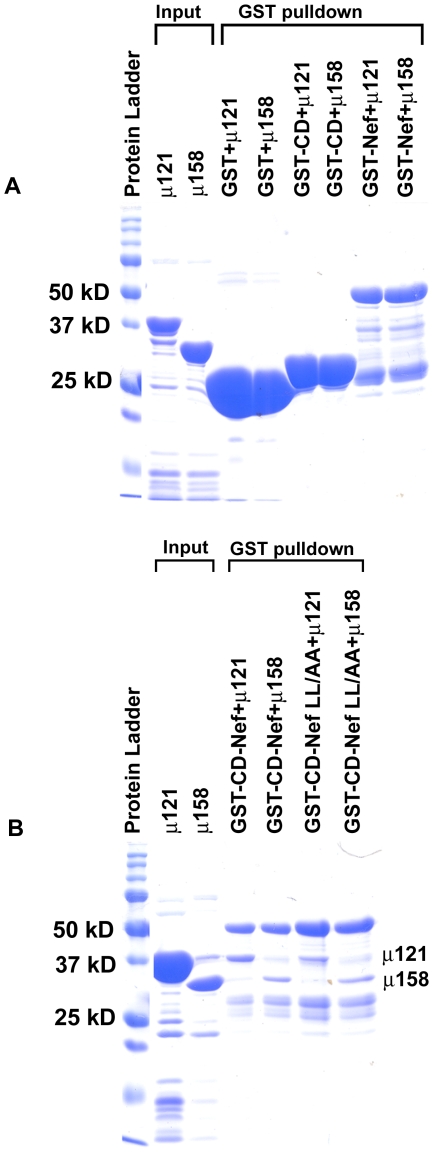
Direct interaction between the MHC-I CD-Nef and μ1: partially purified recombinant proteins were used in GST pulldown assays and analyzed on SDS-PAGE gels by staining with Coomassie blue. (A) From left to right: protein ladder, recombinant μ121 and μ158 proteins as markers, then six lanes of negative controls as indicated. (B) Specific binding between μ1 and MHC-I CD-Nef. Recombinant proteins (“Input”) were run as markers. Right four lanes show binding of μ121 or μ158 with either MHC-I CD-Nef or MHC-I CD-Nef LL/AA. Nef LL/AA indicates alanine substitution of Nef residues L164 and L165.

As described previously [14], the cytoplasmic domain (CD) of MHC-I was fused to the N-terminus of HIV-1 Nef to compensate for the weak interaction between them. This MHC-I CD-Nef chimera was fused to GST (Figure 1) to create a protein for use in pulldown assays with His6-tagged μ1 proteins. For reference, NMR structures of Nef residues 2–57 (PDB code: 1QA5) [15] and residues 56–206 (PDB code: 2NEF) [16] are used. The design of the MHC-I CD-Nef chimera was initially based on studies showing that when the MHC-I α chain was fused to Nef, it co-immunoprecipitated with the AP-1 complex in T-cells [10]. However, the CD of MHC-I has no intrinsic binding activity for the AP-1 complex or for its μ subunit [14]. Therefore, for our *in vitro* binding studies, we used GST, GST-MHC-I CD, and GST-Nef as controls, which as predicted did not bind to the μ1 proteins efficiently (Figure 2A). In contrast, the GST-MHC-I CD-Nef and GST-MHC-I CD-Nef LL/AA bound to the μ121 and the μ158 proteins (Figure 2B) with efficiencies sufficient to enable detection of the interaction directly by Coomassie staining of the protein gels.

In our GST pulldown assays, the binding between MHC-I CD-Nef LL164/165AA and μ1 was comparable to that between MHC-I CD-Nef and μ1 (Figure 2B). This was expected, because although Nef contains a highly conserved dileucine-based adaptor-protein binding motif (ExxxLL), this motif is required for interaction with the γ/σ hemi-complex of AP-1, not for interaction with μ1 [17], [18]. Nef can also bind directly to the μ1 subunit of AP-1 [5], potentially in part through its acidic cluster (E62-65), but this interaction is much weaker than that which occurs when Nef is fused to the CD of MHC-I (Figure 2B and [14]).

These results demonstrate a direct interaction between the μ1 subunit and the fusion protein containing the sequences of the MHC-I CD and Nef. Furthermore, the data recapitulated the cooperative or synergistic nature of the interaction between the MHC-I CD and Nef in binding μ1 [14], since neither the CD nor Nef alone had appreciable binding activity. The data also indicated that the C-terminal two-thirds of μ1 are sufficient for this interaction. Very importantly, the use of purified recombinant proteins expressed in *E. coli* cells suggests that the MHC-I CD and Nef together are sufficient to bind directly to μ1; no additional components or mammalian cellular factors are necessary to reconstitute binding *in vitro*.

### A CD8-MHC-I CD-Nef Chimera Is Down-Regulated Appropriately from the Surface of T Cells

**Figure 3:**
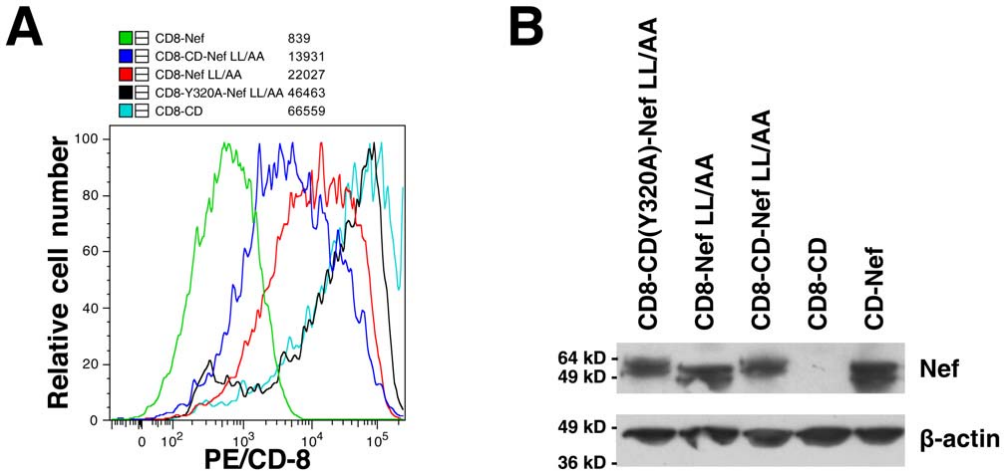
Surface expression in CEM T cells of CD8 chimeras containing the MHC-I CD, Nef, or the MHC-I CD-Nef chimeric sequence as cytoplasmic domains was measured by flow cytometry. Cells were transfected to express GFP as well as the CD8-chimeras, and the cells were gated for low and mid-intensity GFP expression. (A) Relative cell number vs. relative fluorescence intensity of CD8 [phycoerythrin (PE)]. Mean fluorescence intensities for CD8 (PE) are shown. The chimeric MHC-I CD-NefLL/AA sequence directs decreased expression at the cell surface relative to the MHC-I CD alone or NefLL/AA alone. This activity of the MHC-I CD when fused to Nef depends on Y320. (B) The total cellular expression of the chimeric molecules containing Nef was evaluated by western blot. Equal volumes of the transfected cell suspensions were collected before the FACS staining and lysed for western blot analysis using anti-Nef antibody. All chimeric molecules were expressed at a similar level. Actin was probed as a loading control.

To validate the above approach, which relies on an MHC-CD-Nef chimeric protein sequence, we sought evidence that such a chimera would be sorted appropriately within the endosomal system of T cells. To test this, we constructed chimeras in which the MHC-I CD-Nef sequence was fused to the C-terminus of the lumenal and transmembrane domains of CD8. In these constructs, the MHC-I CD-Nef sequence is the cytoplasmic domain of a transmembrane protein that can be detected at the cell surface with an antibody to CD8. We expressed these chimeric proteins in T cells of the CEM line by transient transfection, and then measured their expression at the cell surface using flow cytometry [Figure 3 A, which shows the relative cell number vs. CD8 staining intensity (phycoerythrin) for the mid to low intensity GFP-positive (transfected) cells]. The mean phycoerythrin fluorescence intensities (CD8 surface levels) of the GFP-positive populations indicate that the chimeric MHC-I CD-NefLL/AA sequence decreased the expression at the cell surface by nearly 5-fold relative to the MHC-I CD alone, whereas the NefLL/AA sequence alone decreased the expression at the cell surface by only 3-fold. These date indicate that in the context of the Nef LL164/165AA mutation, which does not affect MHC-I down-regulation under native conditions [19], the fusion of the MHC-I CD to Nef generates a chimeric protein sequence that is recognized in T cells by the endosomal sorting machinery. Furthermore, when we used the CD8-CD (Y320A)-Nef LL/AA chimera, we observed an almost three-fold increase in the expression at the cell surface relative to the CD8-CD-Nef LL/AA chimera in which Y320 is intact. This result validated the role of Y320 in the cytoplasmic tail of MHC-I in the down-regulation of the MHC-I CD-Nef chimera from the cell surface and substantiated its recognition by the endosomal sorting machinery. The total cellular expression of the chimeric proteins containing Nef was checked by western blot using an anti-Nef antibody (Figure 3B). These data indicated that the differences in surface expression detected by flow cytometry were not attributable to differences in the steady state levels of protein expression or to differences in transfection efficiency.

### Key Residues in the MHC-I CD and in Nef Affect the Direct Binding between the CD-Nef Chimera and μ1 *In Vitro*

**Figure 4:**
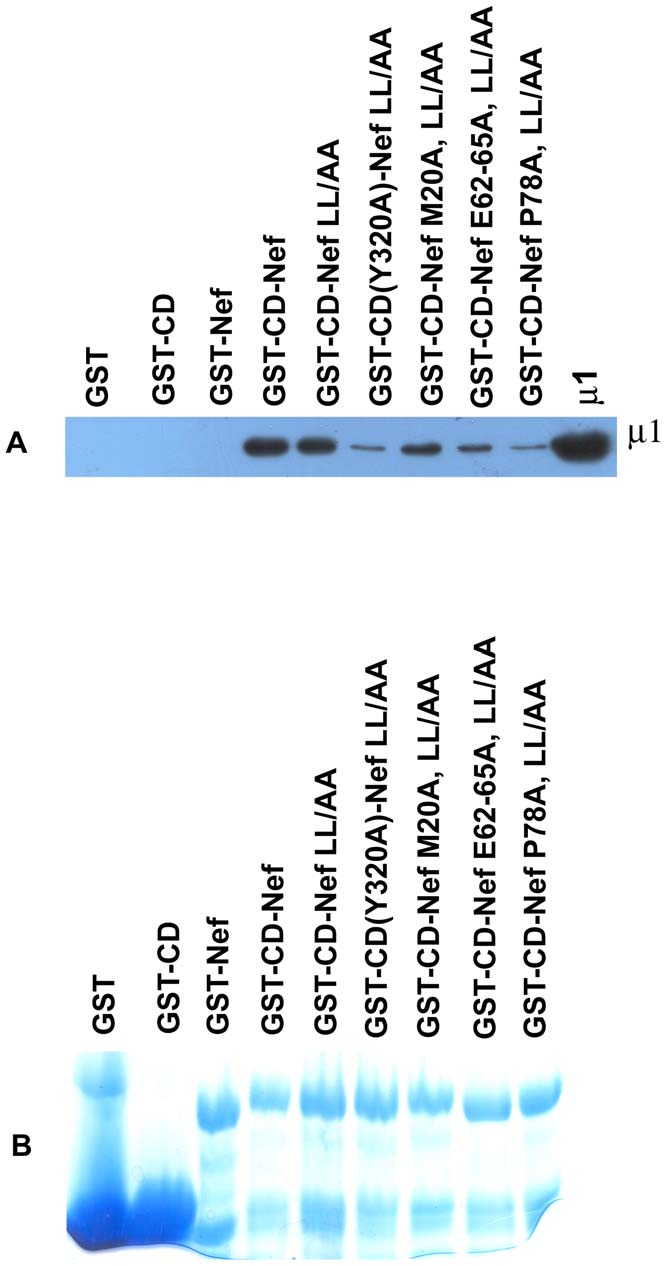
GST pulldown of recombinant μ1 *in vitro* using chimeric proteins in which the MHC-I CD is fused to the N-terminus of Nef and by MHC-I CD-Nef chimera mutants. Various residues within the CD of MHC-I and Nef were mutated to alanine as shown. (A) A western blot of the pulldown was probed with anti-His antibody to detect the recombinant μ1 (μ121). Band quantitation using Image J and setting the pulldown of μ1 by GST-CD-Nef to 100% yielded the following efficiencies for the constructs: GST-CD-NefLL/AA: 77%; GST-CD(Y320A)-NefLL/AA: 16%; GST-CD-Nef M20A,LL/AA: 53%; GST-CD-Nef E62-65A,LL/AA: 30%; GST-CD-Nef P78A,LL/AA: 19%. The results shown are representative of three independent experiments. (B) The amounts of GST-proteins used for binding setup are shown by Coomassie blue staining.

To validate genetically the biological relevance of the direct binding *in vitro* described above, we tested residues in both the MHC-I CD and Nef that are of known importance for the down-regulation of MHC-I by Nef in human cells. These key residues in both the MHC-I CD and Nef are shown by schematic representation (Figure 1). The key residues that have been well characterized previously are Y320 in the MHC-I CD; and M20, the acidic cluster E62-65, and P78 in Nef ([14] and references therein). Although Y320 in the MHC-I CD is within the sequence YSQA and does not conform to the tyrosine-based AP-binding sequence motif (Yxxφ), we and others have previously shown that it likely binds the tyrosine-motif binding pocket of the μ subunit of AP-1 [14], [20]. Here, the mutation of Y320 to alanine substantially affected the binding of the MHC-I CD-Nef chimera to the μ subunit of AP-1 *in vitro* (Figure 4), validating the proposed role of this residue in binding directly to μ1.

We also tested key residues in Nef for their roles in our direct binding assay using recombinant proteins (Figure 4). The Nef residues M20, E62-65 and P78 are of well-validated importance in the down-regulation of MHC-I in human cells ([14] and references therein). Interestingly, M20, which is present in a short α-helical region near the membrane proximal N-terminus of Nef, had little or no effect on the binding of the MHC-I CD-Nef chimera to μ1 in our *in vitro* assay. This could be because the fusion of the N-terminus of Nef to the CD of MHC-I “forces” the association of the two protein sequences, and if Nef M20 is important for this association under native conditions *in vivo*, then its role in the chimera would be rendered moot. Alternatively, Nef M20 could have a role distinct from participation in the ternary interaction of MHC-I, Nef, and AP-1; for example, it could be involved in the trafficking of Nef within the cell. In contrast to M20, Nef residues E62-65 and P78 each contributed substantially to the interaction with μ1 (Figure 4).

As previously proposed [14], the acidic cluster E62-65 of Nef could participate by binding to a basic patch on the μ subunit; μ1 is predicted to be a highly basic protein and its C-terminal two-thirds contains numerous positive charge clusters that could interact electrostatically with the Nef acidic cluster. Previous studies have suggested an alternative mechanism in which Nef down-regulates MHC-I via an interaction of its acidic cluster with PACS-1 (phosphofurin acidic cluster sorting protein-1) [21]. Part of this mechanism would involve an interaction in which PACS-1 connects Nef and AP-1 [21]. In our *in vitro* binding assays, the use of recombinant proteins that are expressed in *E. coli* obviates a role for cellular factors such as PACS-1 in the binding between the MHC-I CD, Nef and μ1. In contrast to the Nef acidic cluster, which could potentially bind a basic patch on μ1, the substantial decrease in binding caused by the P78A mutation has no obvious structural explanation at this time.

Very importantly, mutation of these key residues (Y320 in the CD of MHC-I; and M20, E62-65 and P78 in Nef) suggested that the interactions between the recombinant MHC-I CD-Nef chimera and the recombinant truncated μ1 subunit observed *in vitro* are highly specific. They recapitulate the binding observed using intact AP-1 complexes obtained from human cytoplasm as well as the binding observed using observed full-length μ1 translated *in vitro* [14]. With the exception of Nef M20, all of the mutant proteins with known defects in the down-regulation of MHC-I in human cells had substantial defects in our *in vitro* binding assays using purified recombinant proteins.

### Residues of the μ1 Subunit that Form the Binding Site for Yxxφ Motifs Are Required for Interaction with the MHC-CD-Nef Chimera *In Vitro*

**Figure 5:**
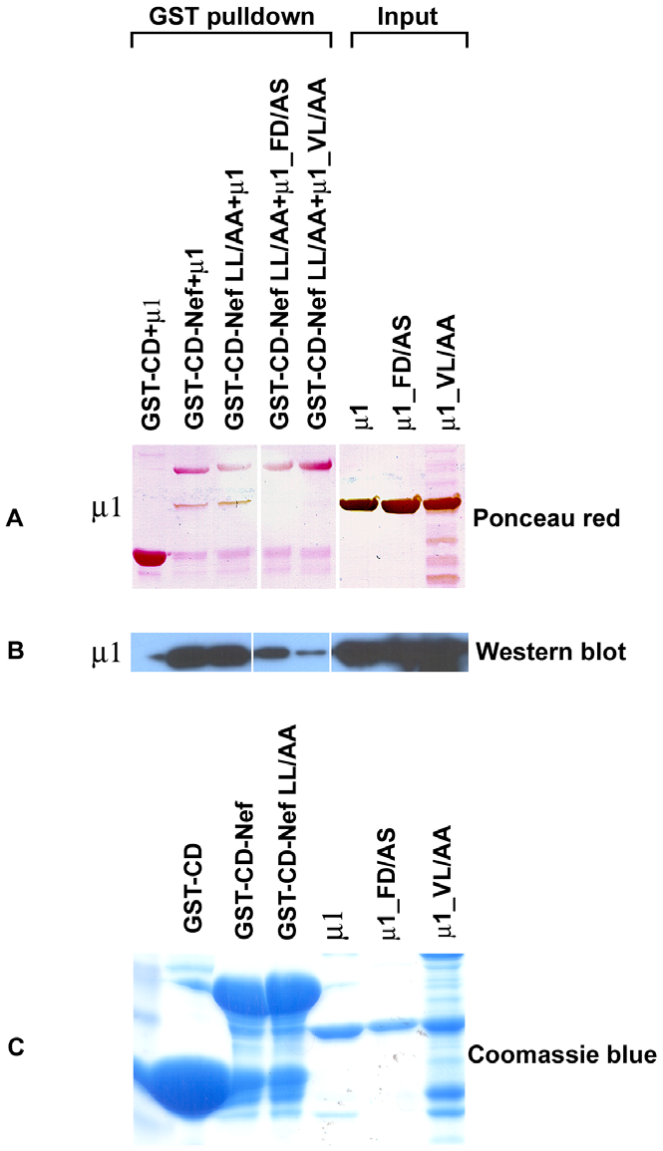
Purified recombinant truncated μ1 and μ1 mutants (F172A/D174S and V392A/L395A) were subjected to GST pulldown assays using MHC-I CD-Nef and MHC-I CD-Nef LL/AA chimeras: (A) Ponceau red staining of PVDF membrane. (B) A western blot of the pulldown was probed with anti-His6 antibody to detect the wild-type μ1 and μ1 mutants. Band quantitation using Image J and setting the pulldown of wild-type μ1 by GST-CD-Nef to 100% yielded the following efficiencies for the μ1 mutants: μ1_FD/AS: 57%; μ1_VL/AA: 24%. The results shown are representative of three independent experiments. (C) The amounts of GST-proteins used for binding setup are shown by Coomassie blue staining.

To further examine the basis of binding between the MHC-I CD-Nef chimera and μ1 and to test specifically the role of the binding pocket on μ1 for tyrosine-based motifs, two mutants of μ1 were made: F172A/D174S and V392A/L395A. Residues F172 and D174 were mutated to disrupt the acceptor site for the tyrosine, whereas V392 and L395 were mutated to disrupt the acceptor site for the Y+3 residue, even though the Y+3 residue in the YSQA MHC-I CD sequence was not expected to bind this hydrophobic pocket. Surprisingly, both of these μ1 mutants showed decreased binding to the MHC-I CD-Nef chimera in comparison to wild-type μ1 (Figure 5). These μ mutants confirm the role and specificity of residues that line the Yxxφ-binding pocket of μ1 in the interaction with MHC-I CD-Nef.

Comparison of the binding sites for tyrosine-based motifs within the μ subunits of AP-2 and AP-1 shows that the structure of the peptide-binding site of μ1 (PDB code: 1W63) [22] is very similar to that of μ2 (PDB code: 1BW8) [11]. In addition, the μ subunit of AP-1 shares ∼40 % sequence identity to that of AP-2. Given the importance of the residues F172/D174 and V392/L395 in binding the MHC-I CD-Nef chimera and the crystal structure of the peptide FYRALM with the μ subunit of AP-2 [11], we predict that residues F172, D174, V392, and V395 in μ1 accommodate the sequence YSQA in the MHC-I CD. Specifically, Y320 of the MHC-I CD likely interacts with F172 and D174 of μ1. In contrast, we think it unlikely that A323 at the Y+3 position in the MHC-I CD contributes directly to binding the hydrophobic pocket. Instead, we favor the hypothesis that a currently unidentified hydrophobic residue in the CD of MHC-I or in Nef makes a key contact with the hydrophobic pocket on μ1 formed by residues V392 and L395. Structural evidence will be required to evaluate these predictions.

### Conclusion

A direct in vitro protein interaction assay using partially purified recombinant proteins produced in *E. coli* demonstrated the formation of a complex between an MHC-I CD-Nef chimeric protein sequence and the C-terminal two-thirds of the μ subunit of AP-1. To validate the relevance of this binding, we tested residues in both the MHC-I CD and Nef that are of known importance for down-regulation in human cells. The tyrosine residue of the YSQA sequence in the MHC-I CD as well as Nef residues E62-65 and P78 each contributed to the interaction, whereas Nef M20 had little to no role. Residues that line the binding pocket on μ1 for Yxxφ motifs, specifically F172 and D174 that form an acceptor site for the tyrosine residue, and V392 and L395 that form an acceptor site for the Y+3 hydrophobic residue, were also required for this interaction. These data indicate that the MHC-I cytoplasmic domain, Nef and the C-terminal two thirds of the μ subunit of AP-1 are sufficient to constitute a biologically relevant interaction. The data also suggest that the hydrophobic pocket on μ1, which typically accommodates the Y+3 residue of Yxxφ motifs, recognizes the MHC-I CD/Nef complex by an as yet undetermined mechanism.
